# Magnetic plasmon resonances in nanostructured topological insulators for strongly enhanced light–MoS_2_ interactions

**DOI:** 10.1038/s41377-020-00429-x

**Published:** 2020-11-23

**Authors:** Hua Lu, Zengji Yue, Yangwu Li, Yinan Zhang, Mingwen Zhang, Wei Zeng, Xuetao Gan, Dong Mao, Fajun Xiao, Ting Mei, Weiyao Zhao, Xiaolin Wang, Min Gu, Jianlin Zhao

**Affiliations:** 1 MOE Key Laboratory of Material Physics and Chemistry under Extraordinary Conditions, and Shaanxi Key Laboratory of Optical Information Technology, School of Physical Science and Technology, Northwestern Polytechnical University, 710129 Xi’an, China; 2 Institute for Superconducting & Electronic Materials and ARC Centre of Excellence in Future Low-Energy Electronics, University of Wollongong, North Wollongong, NSW 2500 Australia; 3 Center for Artificial-Intelligence Nanophotonics, School of Optical-Electrical and Computer Engineering, University of Shanghai for Science and Technology, 200093 Shanghai, China; 4 Guangdong Provincial Key Laboratory of Optical Fiber Sensing and Communications, Institute of Photonics Technology, Jinan University, 510632 Guangzhou, China; 5 State Key Laboratory of Solidification Processing, School of Materials Science and Engineering, Northwestern Polytechnical University, 710072 Xi’an, China

**Keywords:** Nanocavities, Nanophotonics and plasmonics

## Abstract

Magnetic resonances not only play crucial roles in artificial magnetic materials but also offer a promising way for light control and interaction with matter. Recently, magnetic resonance effects have attracted special attention in plasmonic systems for overcoming magnetic response saturation at high frequencies and realizing high-performance optical functionalities. As novel states of matter, topological insulators (TIs) present topologically protected conducting surfaces and insulating bulks in a broad optical range, providing new building blocks for plasmonics. However, until now, high-frequency (e.g. visible range) magnetic resonances and related applications have not been demonstrated in TI systems. Herein, we report for the first time, to our knowledge, a kind of visible range magnetic plasmon resonances (MPRs) in TI structures composed of nanofabricated Sb_2_Te_3_ nanogrooves. The experimental results show that the MPR response can be tailored by adjusting the nanogroove height, width, and pitch, which agrees well with the simulations and theoretical calculations. Moreover, we innovatively integrated monolayer MoS_2_ onto a TI nanostructure and observed strongly reinforced light–MoS_2_ interactions induced by a significant MPR-induced electric field enhancement, remarkable compared with TI-based electric plasmon resonances (EPRs). The MoS_2_ photoluminescence can be flexibly tuned by controlling the incident light polarization. These results enrich TI optical physics and applications in highly efficient optical functionalities as well as artificial magnetic materials at high frequencies.

## Topological insulators: visible range magnetic resonances

Nanostructured antimony telluride (Sb_2_Te_3_) can support visible range magnetic resonances and dramatically enhance the weak interactions of light with 2D materials. Hua Lu and workers from China and Australia used focused ion beam milling to write a grating of periodic nanogrooves into single-crystalline Sb_2_Te_3_, a well-known topological insulator. They then placed a flake of the 2D material MoS_2_ on top. Characterization showed the existence of a kind of magnetic plasmon resonances (MPRs) with a resonant wavelength that redshifts with increasing nanogroove height and pitch and blueshifts with increasing nanogroove width. Visible photoluminescence experiments showed that the MPRs can dramatically increase the emission from the MoS_2_, which could be tuned by changing the polarization angle of the incident excitation light. The findings are expected to aid the development of nanoscale optical devices made from layered nanomaterials.

## Introduction

Artificially structured materials have been broadly used to excite strong magnetic responses for the generation of crucial counterintuitive phenomena, including negative refraction, invisible cloaking, superlensing, etc.^1,2^. To overcome the weak magnetism of natural materials at optical frequencies, metallic molecules (e.g., split rings) with magnetic resonances induced by conduction current loops were proposed to construct artificial magnetic materials with negative refractive indices in the terahertz (THz) and mid-infrared ranges^3,4^. However, transferring magnetic resonances to higher frequencies (especially in the visible range) is restricted by the magnetic response saturation and stringent nanofabrication requirements^5,6^. Fortunately, magnetic resonances with the generation of displacement current loops were observed in specially designed plasmonic nanostructures, such as metallic nanopillar pairs^6^, tailored nanoclusters^7^, and particle-film systems^8,9^. These magnetic plasmon resonances (MPRs) possess excellent capabilities for engineering magnetism, confining light at the nanoscale, and enhancing the optical field in the high-frequency range, thus contributing to promising applications in light manipulation, perfect absorption, sensitive sensing and reinforced light-matter interactions^6–10^. To broaden the actual applications of MPRs, novel materials are currently highly desirable to open new doors for the generation and control of magnetic resonance behavior at optical frequencies. For example, graphene split rings were theoretically predicted to produce a magnetic resonant response stronger than traditional metallic structures, but their operating frequencies were limited to the far-infrared range^11^. Recently, topological insulators (TIs), as new quantum states of matter, have attracted wide attention in electronics, optics, and plasmonics^12–21^. TIs present unconventional conducting edge (or surface) states with topological protection caused by strong spin–orbit coupling of insulating bulk states, distinct from ordinary metals and insulators^12,13^. The time-reversal symmetry of edge (or surface) states with gapless Dirac fermions enables the avoidance of carrier backscattering from non-magnetic impurities^13,14^. The topological edge state was first confirmed in mercury telluride two-dimensional (2D) quantum wells^12^. Afterward, topological surface states with exotic Dirac cones were discovered in three-dimensional (3D) nanomaterials (e.g. Sb_2_Te_3_, Bi_2_Te_3_, and Bi_2_Se_3_)^13,14^. In 2019, thousands of materials were predicted to possess TI properties, paving a prospective path for quantum computing, spintronics, and devices with lower energy consumption^22,23^. Recently, Bi_2_Te_3_, Bi_2_Se_3_, and Bi_1.5_Sb_0.5_Te_1.8_Se_1.2_ TIs have been verified to display ultrahigh refractive indices and light-driven plasmonic activities in an ultrabroad optical range from ultraviolet (UV) to THz^16–21^. MPRs in TIs will be particularly useful for promoting the high-frequency optical activities of TIs and enriching their practical applications, especially in light-matter interactions. However, until now, MPRs and relevant applications have not been reported in TI systems.

Herein, we demonstrate for the first time, to our knowledge, a kind of visible range MPR effect in nanofabricated single-crystalline Sb_2_Te_3_ TI nanogrooves. The experimental results reveal that the MPR response has a particular dependence on the nanogroove height, width, and pitch, consistent with simulations and theoretical calculations. To explore actual applications of TI MPRs, we innovatively integrated monolayer MoS_2_ with a nanostructured TI to improve the intrinsically weak interactions between light and atomic-layer materials. Polarization-dependent photoluminescence (PL) emission was experimentally observed and reasonably analyzed. Benefitting from the strong MPR-induced electric field enhancement, the MoS_2_ PL intensity was remarkably reinforced compared with TI electric plasmon resonances (EPRs). These results will open a new door for exploring novel TI optical physics and applications in optoelectronic devices and artificial magnetic materials.

## Results

### Optical constant and nanostructure of the Sb_2_Te_3_ single crystal

**Fig. 1:**
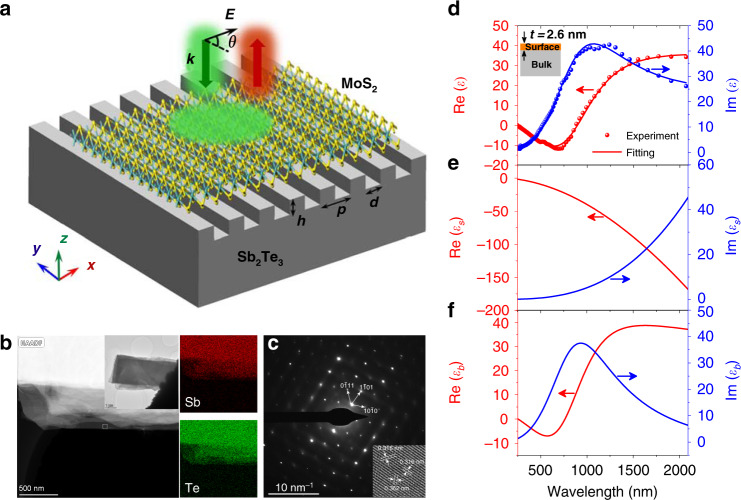
TI nanostructure, material characterization, and optical constant. **a** Schematic of a Sb_2_Te_3_ TI nanogroove grating with height *h*, width *d*, and pitch *p* underneath monolayer MoS_2_. The light is incident on the nanostructure with a polarization angle of *θ*. **b** TEM high-angle annular dark-field (HAADF) image of a part of a thin Sb_2_Te_3_ microflake (inset) on the carbon support film of a copper microgrid. The dotted rectangle denotes the area selected for electron diffraction and high-resolution TEM. The right images are the EDS mapping pictures of the measured part of the Sb_2_Te_3_ microflake for determining the spatial distributions of elements Sb and Te. **c** SAED pattern and HRTEM image (inset) of the Sb_2_Te_3_ single crystal. **d** Measured (circles) and fitted (curves) relative permittivities of the Sb_2_Te_3_ single crystal in the UV, visible, and near-infrared ranges. The fitted real and imaginary permittivities are obtained from the ellipsometer software with a layer-on-bulk model (inset) for the Sb_2_Te_3_ TI. **e**, **f** Real and imaginary permittivities of the conducting surface and insulating bulk.

As shown in Fig. 1a, the TI nanogroove grating structure is fabricated on the surface of a Sb_2_Te_3_ single-crystal film using the focused ion beam (FIB) milling method (see “Materials and methods” section). The height, width, and pitch of the nanogrooves are denoted by *h*, *d*, and *p*, respectively. As a primary member of the 3D TI family, the Sb_2_Te_3_ material possesses distinctly discrepant surface and bulk states, exhibiting excellent optical characteristics^13,15^. Here, the Sb_2_Te_3_ single crystal is grown using the melting and slow-cooling method (see “Materials and methods” section). To clarify the material morphology, we employ transmission electron microscopy (TEM) to obtain the selected area electron diffraction (SAED) pattern and a high-resolution TEM (HRTEM) image of the Sb_2_Te_3_ microflake. The Sb_2_Te_3_ microflake depicted in Fig. 1b is fabricated through mechanical exfoliation and chemical etching methods (Supplementary Methods). As shown in Fig. 1c, the sharp diffraction spots and atomic lattice arrangement verify the hexagonal packed structure of the high-quality Sb_2_Te_3_ single crystal. The chemical composition is confirmed by energy-dispersive X-ray spectroscopy (EDS), revealing that the elemental molar ratio of Sb:Te is 2:3 (Supplementary Fig. S1). The high crystalline quality of Sb_2_Te_3_ can be further verified by the Raman spectrum (Supplementary Fig. S2). The complex relative permittivities of TI materials can be measured by a spectroscopic ellipsometer with considering the surface and bulk states^17^. The conducting surface and insulating bulk can be fitted with the Drude and Tauc–Lorentz dispersion formulas, respectively (Supplementary Methods). Figure 1d shows the fitted relative permittivity of single-crystalline Sb_2_Te_3_ in the UV, visible, and near-infrared ranges, which agrees well with the experimental results. The surface and bulk permittivities (*ε*_s_ and *ε*_b_) are depicted in Fig. 1e, f, respectively. The surface permittivity satisfies the conditions of Re(*ε*_s_) < 0 and −Re(*ε*_s_) > Im(*ε*_s_) at wavelengths from 250 to 2065 nm. This metal-like property of the surface state provides the possibility of generating the plasmonic response at high frequencies^24–26^. The negative permittivity of the bulk state at shorter wavelengths (from 253 to 760 nm) can be attributed to the strong interband electronic absorption similar to semiconductors^17^. Thus, the bulk state can also contribute to the formation of plasmonic resonances at visible wavelengths of less than 760 nm. The surface and bulk states together give rise to the negative permittivity of Sb_2_Te_3_ at wavelengths from 250 to 895 nm, as shown in Fig. 1d. The fitting results (Supplementary Table S1) illustrate that the Sb_2_Te_3_ bulk possesses a bandgap of ~0.33 eV, which is consistent with the reported 0.3 eV^27^. The Sb_2_Te_3_ surface presents an ultrathin layer of 2.6 nm (i.e., *t* = 2.6 nm), similar to the reported 2.5 nm for the TIs in the same family^28^. To the best of our knowledge, this is the first report of surface and bulk optical constants for the Sb_2_Te_3_ single crystal, laying the foundation for exploring Sb_2_Te_3_ optical activities and functionalities.

### Magnetic plasmon resonances in Sb_2_Te_3_ nanostructures

**Fig. 2:**
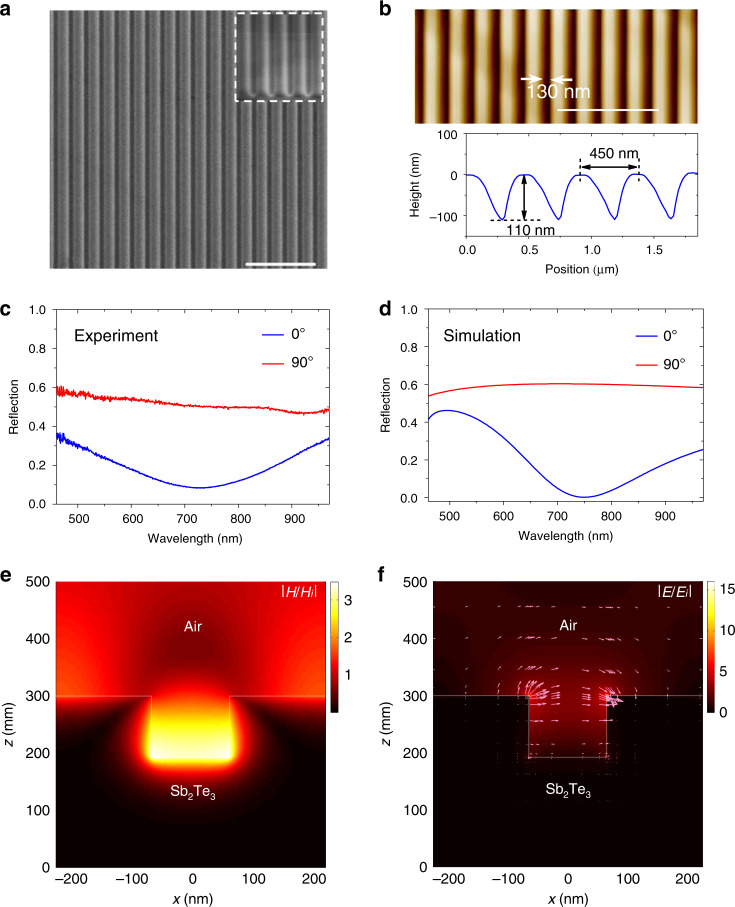
Structure characterization, reflection spectra, and MPR field distribution of TI nanogrooves. **a** Top-view SEM image of the Sb_2_Te_3_ nanogroove grating with *h* = 110 nm, *d* = 130 nm, and *p* = 450 nm. The scale bar is 2 μm. The inset shows an SEM image of Sb_2_Te_3_ nanogrooves with a 52-degree view. **b** Corresponding AFM image of the Sb_2_Te_3_ nanogrooves and the nanogroove height profile along the white line. **c**, **d** Experimentally measured and numerically simulated reflection spectra from the Sb_2_Te_3_ nanogroove grating when *θ* = 0° and 90°. **e** Distribution of the magnetic field |*H*/*H*_i_| in a periodic unit of Sb_2_Te_3_ nanogrooves at the MPR wavelength when *θ* = 0°. **f** Corresponding distribution of electric field |*E*/*E*_i_|. The arrows indicate the directions and relative magnitudes of the electric field. Here, *H*_i_ and *E*_i_ are the magnetic and electric field amplitudes of the incident light, respectively.

First, a nanogroove grating with *h* = 110 nm, *d* = 130 nm, and *p* = 450 nm is fabricated on the Sb_2_Te_3_ film (with a thickness of >300 nm) mechanically exfoliated onto a Si substrate. Scanning electron microscopy (SEM) and atomic force microscopy (AFM) are used to measure the structural profile of the nanogrooves, as shown in Fig. 2a, b, respectively. The reflection spectra from the Sb_2_Te_3_ nanogroove grating are measured using a micro-spectrometer system (see “Materials and methods” section). The experimental results in Fig. 2c reveal that the incident light with polarization perpendicular to the nanogrooves (i.e., *θ* = 0°) possesses a distinct reflection dip at ~736 nm. However, the reflection dip disappears when the polarization is parallel to the nanogrooves (i.e., *θ* = 90°). The finite-difference time-domain (FDTD) numerical simulations (see “Materials and methods” section) agree well with the experimental results, as depicted in Fig. 2d. To clarify the reflection mechanism, we plot the magnetic and electric field distributions at the reflection dip when *θ* = 0°, as shown in Fig. 2e, f, respectively. Interestingly, the magnetic field energies are enhanced and are mainly concentrated in the Sb_2_Te_3_ nanogrooves, exhibiting a strong diamagnetic effect. In Fig. 2f, the arrows indicate the direction and amplitude of the electric field vector, revealing the generation of displacement current loops in the nanogrooves. The electric current along the nanogroove surface is excited by the magnetic field component parallel to the Sb_2_Te_3_ nanogroove (Supplementary Fig. S3). The visible range resonance generated in this split-ring-like TI nanostructure is regarded as a typical magnetic resonance similar to MPR^29^. When the magnetic field component of incident light is perpendicular to the nanogrooves (i.e., *θ* = 90°), the magnetic resonance cannot be effectively excited, restraining the appearance of the reflection dip. Under the resonant condition, the TI surface charges accumulate at the upper corners of the nanogrooves, accompanied by a strong electric field intensity (|*E*/*E*_i_|^2^) enhancement of >200-fold, as depicted in Fig. 2f. From Fig. 2c, we find that TIs can present a broader MPR (or MPR-like) spectrum than metals. The broad MPR spectrum contributes to a relatively large wavelength range for strong field enhancement. The significant field enhancement in nanostructured TIs will open a new door for the improvement of nanoscale light-matter interactions. It is worth noting that the TI surface layer contributes to the redshift and narrowing of the resonant spectrum^17^ (Supplementary Fig. S4a). At the MPR wavelength, the TI surface and bulk present absorption peak values of ~6% and ~94%, respectively (Supplementary Fig. S4b).

**Fig. 3:**
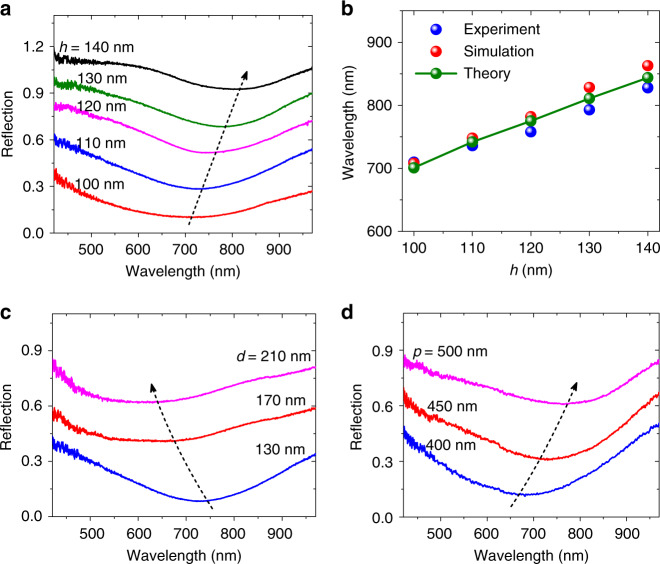
Measured MPR spectral response of TI nanogrooves with different structural parameters. **a** Experimentally measured reflection spectra from the Sb_2_Te_3_ nanogroove grating with different heights *h* = 100, 110, 120, 130, and 140 nm when *d* = 130 nm, *p* = 450 nm, and *θ* = 0°. **b** Corresponding MPR wavelengths of Sb_2_Te_3_ nanogrooves with different *h*. The experimental, simulation, and theoretical results are obtained by the micro-spectrometer, FDTD method, and MLC circuit model, respectively. **c**, **d** Experimentally measured reflection spectra from the Sb_2_Te_3_ nanogroove grating with different widths *d* = 130, 170, and 210 nm, when *h* = 110 nm, *p* = 450 nm, and *θ* = 0° and with different pitches *p* = 400, 450, and 500 nm, when *h* = 120 nm, *d* = 170 nm, and *θ* = 0°.

Subsequently, we investigate the dependence of the magnetic resonance on the structural parameters (i.e., height *h*, width *d*, and pitch *p*) of the Sb_2_Te_3_ TI nanogrooves. Figure 3a shows that the reflection dip presents a distinct redshift with increasing *h* when *θ* = 0°. The MPR wavelength has a nearly linear relationship with *h* when *p* = 450 nm and *d* = 130 nm, as depicted in Fig. 3b. The experimental results are in excellent agreement with the numerical simulations. The MPRs can be reasonably analyzed by a mutual inductor–inductor–capacitor (MLC) circuit model^30^. The MPR wavelength can be described as *λ*_*M*_ = 2*πc*[(*L* + *M*)*C*]^0.5^, where *L*, *M*, and *C* are the inductance containing the nanogroove ridge inductance/kinetic inductance, the mutual inductance between two adjacent circuits, and the capacitance between two nanogroove ridges (Supplementary Methods). According to the MLC circuit model, we can theoretically deduce the MPR wavelength in the Sb_2_Te_3_ nanogrooves. The theoretical calculations are well consistent with the experimental and simulation results, as shown in Fig. 3b. The MLC circuit model indicates that the MPR response is also dependent on the nanogroove width *d*. Here, we measure the reflection spectra from the Sb_2_Te_3_ nanogroove gratings with different *d* when *h* = 110 nm and *p* = 450 nm. The experimental results in Fig. 3c demonstrate that the MPR wavelength exhibits a blueshift as *d* increases, which agrees well with the simulations (Supplementary Fig. S5). We also find that the MPR response also depends on the pitch of the nanogroove grating. The experiments in Fig. 3d show that the MPR wavelength redshifts with increasing *p*, in accordance with the simulations (Supplementary Fig. S5). From the MLC circuit model, we can see that the mutual inductance *M* increases with *p*, giving rise to the redshift of *λ*_*M*_. The nanogroove height, width, and pitch are the effective parameters for the tuning and selection of MPR wavelengths in TIs.

### PL emission from the monolayer MoS_2_/Sb_2_Te_3_ nanogroove heterostructure

**Fig. 4:**
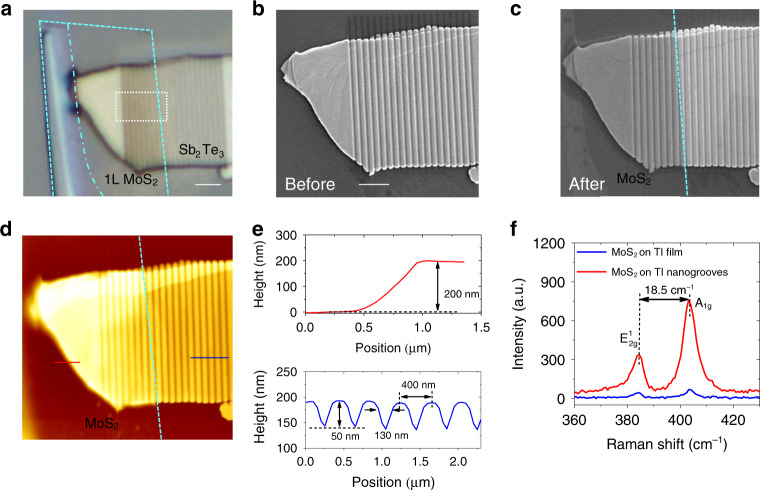
Structure and material characterization of the monolayer MoS_2_/Sb_2_Te_3_ nanogroove heterostructure. **a** Optical microscope image of Sb_2_Te_3_ nanogrooves with a transferred MoS_2_ layer. The area marked by the cyan dashed line denotes the MoS_2_ layer. The area on the right side of the dashed-dotted line represents monolayer (1 L) MoS_2_. The scale bar is 2 μm. **b**, **c** SEM images of Sb_2_Te_3_ nanogrooves before and after transferring MoS_2_. The scale bar is 2 μm in **b**. **d** AFM image of Sb_2_Te_3_ nanogrooves with MoS_2_. **e** AFM-measured height profiles of the Sb_2_Te_3_ film and nanogrooves at the positions along the red and blue lines in **d**. **f** Raman spectra of the MoS_2_ layer on the Sb_2_Te_3_ TI film and nanogrooves excited with the 532 nm laser when *θ* = 0°.

**Fig. 5:**
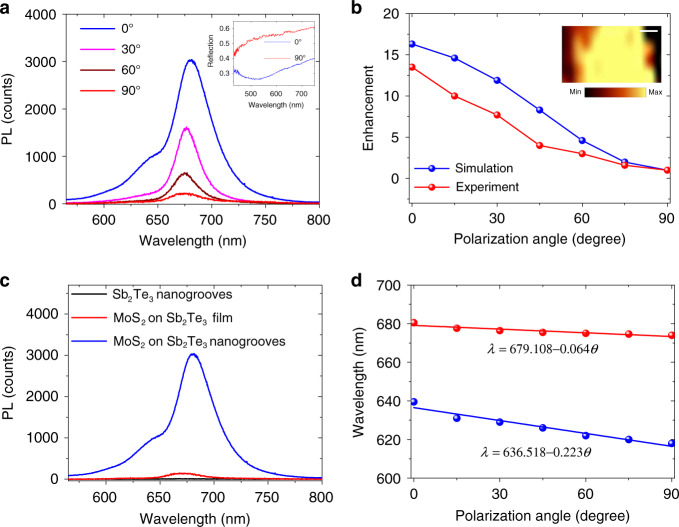
PL emission response of the monolayer MoS_2_/Sb_2_Te_3_ nanogroove heterostructure. **a** PL emission spectra of the monolayer MoS_2_/Sb_2_Te_3_ nanogroove heterostructure for different incident polarization angles *θ*. The inset shows the reflection spectra of Sb_2_Te_3_ nanogrooves with monolayer MoS_2_ when *θ* = 0° and 90°. **b** Enhancement factors of the integrated electric field intensity in Sb_2_Te_3_ nanogrooves at 532 nm and PL emission at the A excitonic peak of monolayer MoS_2_ for different *θ* compared to the case of *θ* = 90°. The inset shows the PL intensity map spectrally integrated in the range of 665–695 nm when *θ* = 0° in the nanogroove area marked as the white dashed square frame in Fig. 4a. The scale bar is 1 μm. **c** PL emission spectra from the Sb_2_Te_3_ nanogrooves and monolayer MoS_2_ on the Sb_2_Te_3_ film/nanogrooves. **d** Dependence of A and B excitonic peak wavelengths on *θ*.

As mentioned above, the magnetic resonance in the TI nanostructure with strong field enhancement may offer a promising route for boosting light-matter interactions. Transition metal dichalcogenides (TMDCs), as a kind of 2D materials with unique semiconductor-like band structures, are regarded as a favorable platform for advancing next-generation optoelectronics due to the unique electric, mechanical and optical properties^31,32^. As a prototypical TMDC semiconductor, MoS_2_ possesses a state transition from an indirect bandgap of 1.2 eV to a direct bandgap of 1.8 eV as it transforms from bulk to monolayer^31,33^. The photoemission, excitonic binding, and chemical stability of monolayer MoS_2_ have drawn wide attention for the PL emission, promoting the achievement of atomically thin active light emitters and sources^33–35^. However, the atomically thin layer with poor light-matter interactions hinders the substantial application of MoS_2_ for light harvesting and emission^33,35^. Here, we innovatively integrate a MoS_2_ atomic monolayer with a Sb_2_Te_3_ TI nanostructure to explore the tailoring and enhancement of PL emission. As depicted in Figs. 1a and 4a, a MoS_2_ flake is mechanically exfoliated and coated on the Sb_2_Te_3_ nanogrooves using the fixed-point transfer method (see “Materials and methods” section). Figure 4b, c shows SEM images of the Sb_2_Te_3_ nanostructure before and after transferring the MoS_2_ flake, respectively. We can see that the MoS_2_ layer is precisely transferred onto the Sb_2_Te_3_ film with fabricated nanogrooves. The nanogroove grating is fabricated with *h* = 50 nm, *d* = 130 nm, and *p* = 400 nm on the Sb_2_Te_3_ film with a thickness of ~200 nm, as shown in Fig. 4d, e. The Raman spectrum in Fig. 4f shows that the in-plane vibrational mode $E^1_{2g}$ and out-of-plane vibrational mode A_1*g*_ can be excited when a 532 nm laser beam impinges onto the MoS_2_ layer over Sb_2_Te_3_. The frequency difference between the $E^1_{2g}$ and A_1*g*_ modes is ~18.5 cm^−^^1^, which agrees well with the reported value of monolayer MoS_2_^34^. As depicted in the inset of Fig. 5a, the Sb_2_Te_3_ nanogroove grating with monolayer MoS_2_ presents a reflection dip at ~532 nm when *θ* = 0° while retaining a high reflection when *θ* = 90°. This illustrates that the magnetic resonance can be generated at ~532 nm in the monolayer MoS_2_/Sb_2_Te_3_ nanogroove heterostructure. The numerical simulations are consistent with the experimental results (Supplementary Fig. S6). To reveal the light–MoS_2_ interaction enhancement, we demonstrate the luminescence emission response in the monolayer MoS_2_/Sb_2_Te_3_ nanogroove heterostructure using a confocal micro-spectrometry system with an excitation wavelength of 532 nm. Figure 5a displays the PL intensity spectra of the heterostructure for different incident polarization angles. Two PL peaks occur at 674.5 and 618.0 nm when *θ* = 90°, as can be more clearly seen in Supplementary Fig. S7. The inset of Fig. 5b depicts the PL intensity map spectrally integrated from 665 to 695 nm in the dashed square frame of Fig. 4a when *θ* = 0°. We can see that the Sb_2_Te_3_ nanogrooves with MoS_2_ can effectively excite the PL signal, while the Sb_2_Te_3_ nanogrooves cannot solely emit upon photoexcitation, as shown in Fig. 5c. The above two PL peaks correspond to the positions of the A and B direct excitonic transitions for monolayer MoS_2_ at the K point of the Brillouin zone^36^. The two resonances derive from the energy splitting from spin–orbit coupling of the valence band in monolayer MoS_2_^36–38^. The MoS_2_ PL emission is sensitive to the substrate. The PL emission intensity of MoS_2_ on SiO_2_ will be stronger than that of MoS_2_ on the TI film. As shown in Fig. 5a, the intensities of the PL peaks drastically increase with the decrease in *θ* from 90° to 0°. The PL emission intensity with *θ* = 0° is particularly enhanced compared with the case of *θ* = 90°. Actually, the PL emission improvement is mainly dependent on the electric field enhancement in luminescent materials^21,31,37^. To clarify the mechanism of PL reinforcement, we numerically calculate the integrated electric field intensity in the Sb_2_Te_3_ nanogrooves. In contrast with the electric field with *θ* = 90°, we find that the enhancement factor of the integrated electric field intensity at 532 nm monotonically decreases as *θ* increases from 0° to 90°. As shown in Fig. 5b, the enhancement factor of the electric field intensity is close to the reinforced strength of the MoS_2_ PL excitonic peak. Therefore, the electric field enhancement plays a critical role in the PL reinforcement. In addition, the MoS_2_ PL emission is influenced by the support structures. The low reflection at the exciton wavelength may weaken the detected PL emission (Supplementary Fig. S8). Moreover, the PL peak wavelengths exhibit a linear redshift with decreasing *θ*, as depicted in Fig. 5d. The shifts satisfy the relations *λ* = 679.108–0.064*θ* and *λ* = 636.518–0.223*θ* for the two PL peaks. When *θ* = 0°, the PL peaks can approach the 680.5 and 638.0 nm wavelengths. Here, the redshift of the PL peak can be attributed to the generation of trions (a type of quasi-particle state) in monolayer MoS_2_ induced by the doping of MPR-excited hot electrons^37,38^. The A− trion, neutral A exciton, and B exciton in MoS_2_ can be extracted by fitting the PL spectrum using the multi-Lorentzian fitting method^38^. The MoS_2_ A− trion, A exciton, and B exciton peaks approximately localize at 687.4, 673.2, and 618.0 nm (i.e., 1.80, 1.84, and 2.01 eV) in the heterostructure with *θ* = 90°, respectively (Supplementary Fig. S7). The A exciton dominates the MoS_2_ PL emission when *θ* = 90° and decays with decreasing *θ*, while the A− trion increases gradually. This may stem from the higher hot-electron doping in MoS_2_ induced by the stronger magnetic resonance in the nanogrooves with smaller *θ*^38^. This phenomenon confirms the exciton-trion competition in plasmon systems^37^. The MPR-induced field enhancement and exciton-trion competition result in the different portions of the A exciton and A-trion/B exciton when *θ* changes from 90° to 0° (Supplementary Fig. S7). The appearance of hot electron-induced trions may also cause the reduction of the PL enhancement^37^. The energy shift of the A peak is 20 meV when *θ* changes from 90° to 0°, identical to the binding energy of trions in monolayer MoS_2_^38^. The dependence of the PL emission height and position on the incident polarization offers a controllable scheme for tailoring light–MoS_2_ interactions in artificial nanostructures. As depicted in Fig. 5c, the PL emission of monolayer MoS_2_ based on MPR shows a strong reinforcement of 21-fold, a remarkable value compared with that of EPRs on TI nanoplates^21^. It should be noted that the PL emission reinforcement mainly results from the enhancement of the integrated electric field intensity in the laser impingement area. Therefore, the reinforcement of the PL intensity is lower than that of the electric field in Fig. 2f. As shown in Fig. 4f, the Raman signal can be improved by one order of magnitude with the generation of magnetic resonance.

## Discussion

In this article, the topological optical features of a Sb_2_Te_3_ single crystal have been experimentally demonstrated. The results show that the surface and bulk states of the Sb_2_Te_3_ TI exhibit obvious metal- and semiconductor-like characteristics in the range from UV to near-infrared, respectively, enabling plasmonic excitation at high frequencies. Visible range MPR-like magnetic resonances were first observed in Sb_2_Te_3_ nanogrooves nanofabricated using the FIB milling method, breaking through the research limitation of TI EPRs^16–21^. Both the experimental and simulation results indicate that this MPR response particularly depends on the structural parameters. More specifically, the MPR wavelength redshifts with increasing nanogroove height and pitch, while it presents a blueshift with increasing nanogroove width. The MPR behavior can be effectively analyzed by the MLC circuit theoretical model. To explore potential applications of this MPR effect, we have integrated an advanced 2D nanomaterial (i.e., monolayer MoS_2_) with TI nanostructures to boost the light-matter interactions as an example. The experimental results show that the PL emission of monolayer MoS_2_ can be dramatically reinforced with MPR generation, breaking the intrinsic limitation of the poor interaction between light and atomically thin materials. The peak intensities and wavelengths of the MoS_2_ PL can be tuned by adjusting the polarization angle of the incident light, which can be attributed to the resonance-induced polarization-dependent electric field enhancement and generation of the A− trion. The MoS_2_ PL emission can be reinforced by 21-fold based on the MPRs in TI nanostructures, remarkably compared with TI-based EPRs^21^. The conventional Au material can also effectively promote the PL emission of MoS_2_^39^. TIs can support plasmons at wavelengths from UV to THz^16–21^, making the generation of electric/magnetic resonances in an ultrabroad range possible. The excited wavelengths for Au-based PL enhancement can be extended by TI-based plasmons. The resonant field enhancement in the TI nanogrooves may not be the highest value, but the resonance spectral width with strong field enhancement is particularly broad. When the resonance wavelength is located around the MoS_2_ PL peak position, the electric field at 532 nm can still be enhanced by >100 times, which results in obvious MoS_2_ PL reinforcement (Supplementary Fig. S9). We find that the field enhancement can be further promoted through structural modification (Supplementary Fig. S10), enabling more applications of TIs in optical devices, such as nanoscale light sources, nonlinear frequency converters, and light-harvesting elements. This kind of magnetic resonance can also be generated in other TIs, such as Bi_2_Te_3_ and Bi_2_Se_3_. The various structures, such as nanopillars^6^, nanoclusters^7^, nanoparticles on film^8,9^, and nanocups^10^, will further enrich MPR effects in TI systems. These results not only enrich TI optical physics but also advance applications of TIs in optoelectronic devices and artificial magnetic materials.

## Materials and methods

### Growth of the Sb_2_Te_3_ single crystal

The high-quality Sb_2_Te_3_ single crystal is grown using the melting and slow-cooling method. High-purity Sb and Te powders with an atomic ratio of 2:3, as the starting materials, are sealed in a quartz tube. The crystals can be grown in a vertical furnace according to the following procedures: (1) The Sb and Te mixed powders are heated to 900 °C and completely melt. (2) The temperature is reduced quickly to 650 °C at a rate of 60 °C/h and then slowly to 550 °C at a rate of 2 °C/h. (3) The mixture is naturally cooled to room temperature. The Sb_2_Te_3_ single-crystalline character and stoichiometry can be confirmed by TEM, Raman, and EDS characterization.

### Fabrication of Sb_2_Te_3_ nanostructures and transfer of MoS_2_

The Sb_2_Te_3_ film is mechanically exfoliated on a Si substrate using “Scotch” tape from the Sb_2_Te_3_ single crystal. The nanogroove grating is fabricated on the Sb_2_Te_3_ film using a FIB milling system (FEI Helios G4 CX) with a 30 kV voltage and a 7.7 pA current. The beam current should be controlled at a relatively low level for robust FIB fabrication. The MoS_2_ flakes are fabricated by exfoliating them from the MoS_2_ bulk material, repeatedly peeling them off with tape, and sticking them on a polydimethylsiloxane (PDMS) film. If a MoS_2_ flake on the PDMS has the most transparent area of several microns in size, then the MoS_2_ flake is transferred onto the Sb_2_Te_3_ nanogrooves using an optical microscope/micromanipulation system. Thus, the dry fixed-point transfer of MoS_2_ is completed.

### Characterization of materials and nanostructures

The electron diffraction pattern and high-resolution TEM image of the Sb_2_Te_3_ single crystal are obtained by TEM equipment (FEI Talos F200X) with a voltage of 200 kV. Raman spectra of MoS_2_ and Sb_2_Te_3_, as well as PL emission spectra, are acquired using confocal micro-spectrometry (WITec Alpha 300R) with a linearly polarized 532 nm laser and an adjustable beam size (minimum diameter: 400 nm). SEM images of Sb_2_Te_3_ nanostructures are acquired by SEM equipment integrated with the FIB (FEI Helios G4 CX) using a 5 kV voltage and a 21 pA current. To avoid damaging the TI material, the beam current should not be too high (<43 pA) for SEM imaging. AFM images and height profiles of Sb_2_Te_3_ nanostructures are obtained by a commercial AFM system (Bruker). The relative permittivity of the Sb_2_Te_3_ single crystal in the UV, visible and near-infrared ranges is measured by a spectroscopic ellipsometer (HORIBA) with an angle of 70° for incident light. Reflection spectra from Sb_2_Te_3_ nanogrooves with/without MoS_2_ are measured by a home-made micro-spectrometer with a white light source impinging on the sample through a microscope and then reflected onto a CCD camera with a spectrometer (Andor).

### Numerical simulations

The reflection spectra and field distributions of Sb_2_Te_3_ nanostructures are numerically simulated using the FDTD method^20,40,41^. The perfectly matched layer absorbing boundary condition and periodic boundary condition are set at the top/bottom and left/right sides of the computational space, respectively. A non-uniform mesh is employed in the *x* and *z* axis directions of TI nanostructures. The maximum mesh steps of the Sb_2_Te_3_ surface layers and monolayer MoS_2_ (0.615 nm) are set as 0.3 nm and 0.1 nm, respectively. The maximum mesh step of the Sb_2_Te_3_ bulk layer, air, and Si substrate is set as 5 nm. The relative permittivities in Fig. 1e, f are set for the surface and bulk states of the Sb_2_Te_3_ TI, respectively. The complex relative permittivity of the Si substrate is achieved from experimental data^42^. The relative permittivity of monolayer MoS_2_ measured by Li et al. is used in the simulations^43^. The reflection spectra are calculated using *R* = |*P*_r_/*P*_i_|, where *P*_i_ and *P*_r_ are the light powers incident on and reflected from the Sb_2_Te_3_ nanostructures, respectively.

## Supplementary information

Supplementary information

## Data Availability

The data sets generated and analyzed in the article are available from the corresponding authors upon reasonable request.
