# Supplementary material for: Magnetic plasmon resonances in nanostructured topological insulators for strongly enhanced light–MoS2 interactions
Source: Light Sci Appl. 2020 Nov 23;9:191. doi: 10.1038/s41377-020-00429-x (PMC7680790; doi:10.1038/s41377-020-00429-x)
Supplement: Supplementary file 1 — Supplementary information [file 41377_2020_429_MOESM1_ESM.pdf]

Supplementary information for

Magnetic plasmon resonances in nanostructured topological insulators for strongly enhanced light-MoS_2 interactions

Hua Lu,[1,*] Zengji Yue,[2] Yangwu Li,[1] Yinan Zhang,[3,4*] Mingwen Zhang,[1] Wei Zeng,[5] Xuetao Gan,[1] Dong Mao,[1] Fajun Xiao,[1] Ting Mei,[1] Weiyao Zhao,[2] Xiaolin Wang,[2] Min Gu[3] and Jianlin Zhao[1,*]

[1]*MOE Key Laboratory of Material Physics and Chemistry under Extraordinary Conditions, and Shaanxi Key Laboratory of Optical Information Technology, School of Physical Science and Technology, Northwestern Polytechnical University, Xi'an, 710129, China*

[2]*Institute for Superconducting & Electronic Materials and ARC Centre of Excellence in Future Low-Energy Electronics, University of Wollongong, North Wollongong, New South Wales, 2500, Australia*

[3]*Center for Artificial-Intelligence Nanophotonics, School of Optical-Electrical and Computer Engineering, University of Shanghai for Science and Technology, Shanghai, 200093, China*

[4]*Guangdong Provincial Key Laboratory of Optical Fiber Sensing and Communications, Institute of Photonics Technology, Jinan University, Guangzhou, 510632, China*

[5]*State Key Laboratory of Solidification Processing, School of Materials Science and Engineering, Northwestern Polytechnical University, Xi'an, 710072, China*

Correspondence: Hua Lu (hualu@nwpu.edu.cn), Yinan Zhang (zhangyinan@jnu.edu.cn) or Jianlin Zhao (jlzhao@nwpu.edu.cn).

This file includes:

Supplementary Figures S1 to S11

Supplementary Table S1

Supplementary Methods

Supplementary Figures

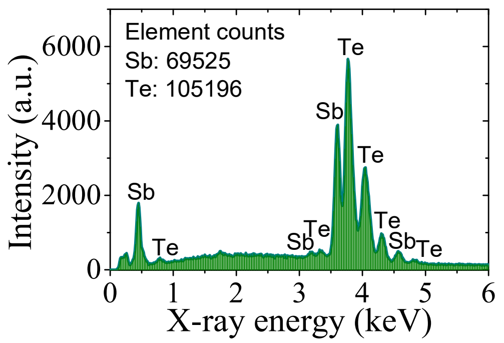

Fig. S1 EDS measurement of Sb_2Te_3 single crystal. Energy dispersive X-ray spectrum of Sb_2Te_3 single crystal measured by using energy dispersive spectrometer (EDS) integrated with the SEM. The element counts reveal that the elemental molar ratio of Sb:Te is about 2:3.

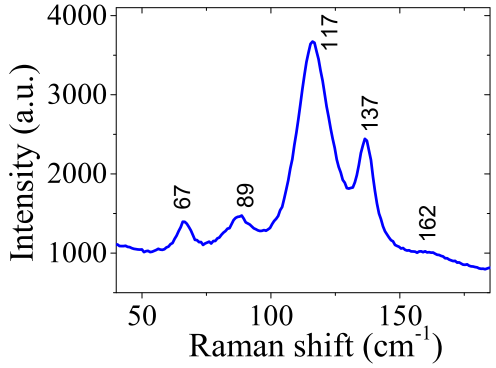

Fig. S2 Raman spectrum of Sb_2Te_3 single crystal. Raman spectrum of Sb_2Te_3 single crystal with an excitation wavelength of 532 nm. The Raman spectral peaks locate at 67, 89, 117, 137 and 162 cm^{-1}, which agrees well with the reported results of Sb_2Te_3 single crystal[S1].

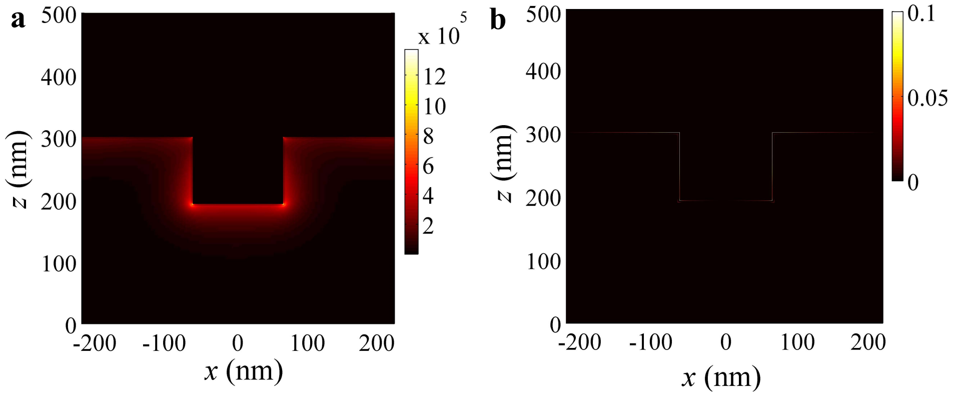

Fig. S3 Current and charge density distributions. **a** Current and **b** charge density distributions in the Sb_2Te_3 nanogrooves at the MPR wavelength when h=110 nm, d=130 nm and p=450 nm.

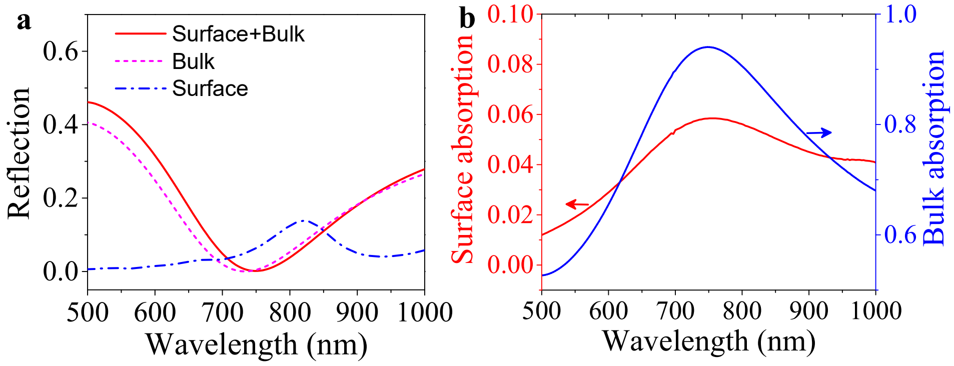

Fig. S4 TI surface and bulk response for MPR in Sb_2Te_3 nanogrooves. a Reflection spectra of Sb_2Te_3 TI nanogroove structures with surface and bulk layers, with only bulk layer and with only surface layer. **b** Absorption spectra of TI surface and bulk in the nanogroove structure. Here, the geometrical parameters are set as h=110 nm, d=130 nm and p=450 nm. The results illustrate that this magnetic resonance in TIs can be attributed to not only the metal-like surface with plasmonic properties but also the lossy semiconductor-like bulk (namely, the joint action of TI surface and bulk). It is different from the traditional MPRs in metallic structures, which mainly results from the metals with plasmonic properties. With considering the magnetic response similar to MPRs in metallic u-shaped structures[S2], we can also call it MPR-like effect.

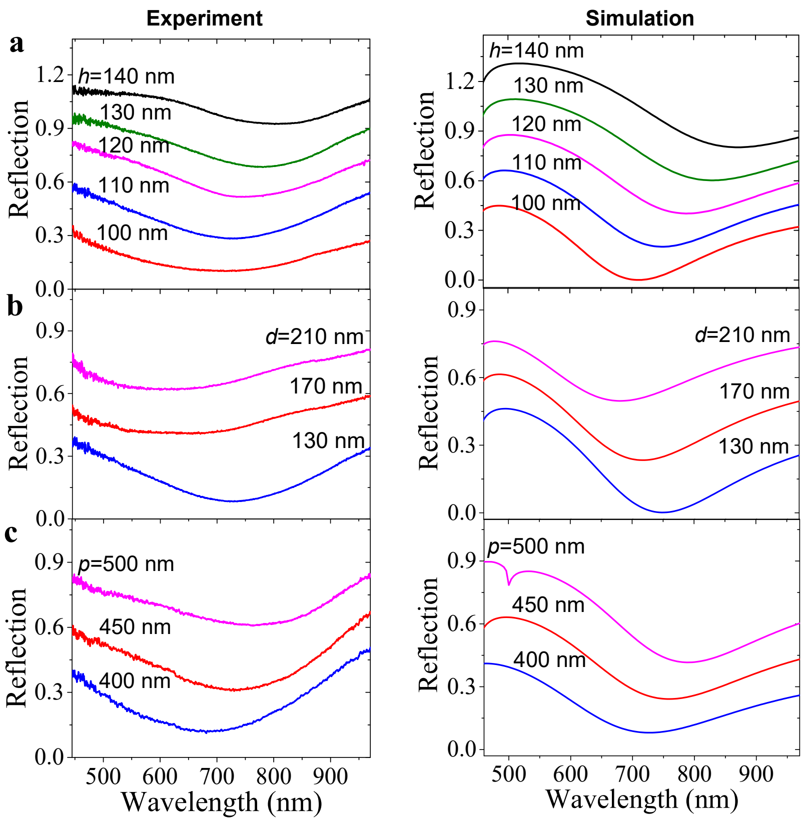

Fig. S5 Experiment and simulation results of reflection spectra from the Sb_2Te_3 nanogroove structures. Experimentally measured and numerically simulated reflection spectra from the Sb_2Te_3 nanogrooves with h=100, 110, 120, 130 and 140 nm when d=130 nm, p=450 nm and θ=0° **a**, with d=130, 170 and 210 nm when h=110 nm, p=450 nm and θ=0° **b** and with p=400, 450 and 500 nm when h=120 nm, d=170 nm and θ=0° **c**.

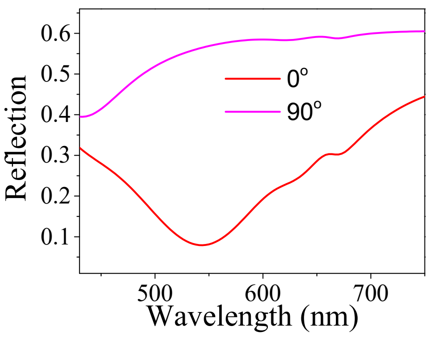

Fig. S6 Simulated reflection spectra of Sb_2Te_3 nanogrooves with MoS_2. Numerically simulated reflection spectra from the monolayer MoS_2/Sb_2Te_3 nanogroove heterostructure in Fig. 4a when θ=0° and 90°.

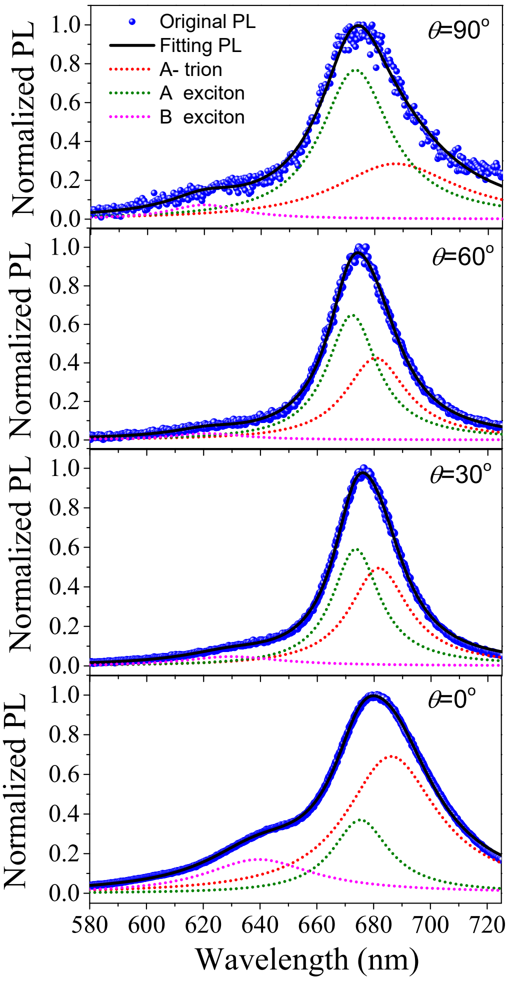

Fig. S7 Decomposed PL spectra to trions and excitons under different incident polarizations. Decomposed normalized PL spectra into the A- trion, neutral A exciton and B exciton under different incident polarization angles θ.

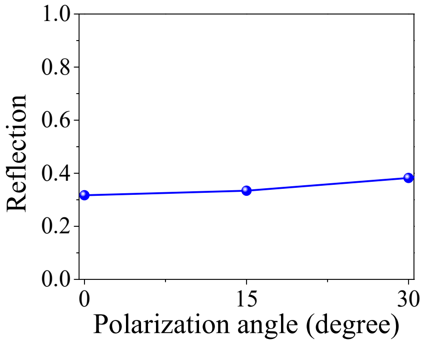

Fig. S8 Reflection at the A excitonic peak wavelength of MoS_2 PL. Reflections of the monolayer MoS_2/Sb_2Te_3 nanogroove heterostructure at the A excitonic peak wavelength of MoS_2 PL when θ=0°, 15° and 30°.

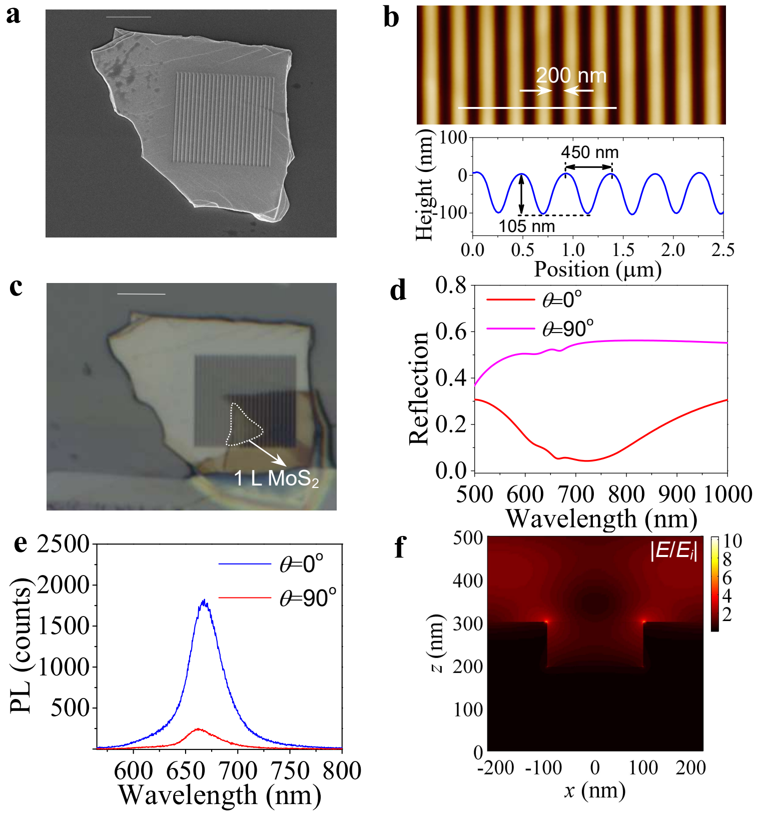

Fig. S9 PL emission and field enhancement of the MoS_2/Sb_2Te_3 nanogroove structure with *h*=105 nm, *d*=200 nm and *p*=450 nm. a SEM image of the Sb_2Te_3 nanogroove structure with *h*=105 nm, *d*=200 nm and *p*=450 nm. The scale bar is 5 μm. **b** AFM image of the nanogrooves and corresponding height profile along the white line. **c** Optical microscope image of the nanogroove structure with transferred MoS_2 layer. The area marked by the white dashed line represents monolayer (1L) MoS_2. The scale bar is 5 μm. **d** Reflection spectra of the nanogroove structure with monolayer MoS_2 when θ=0° and 90°. **e** PL emission spectra of monolayer MoS_2 on the nanogrooves excited with the 532 nm laser when θ=0° and 90°. **f** Distribution of electric field $|E/E_i|$ at the 532 nm wavelength in the Sb_2Te_3 nanogroove structure.

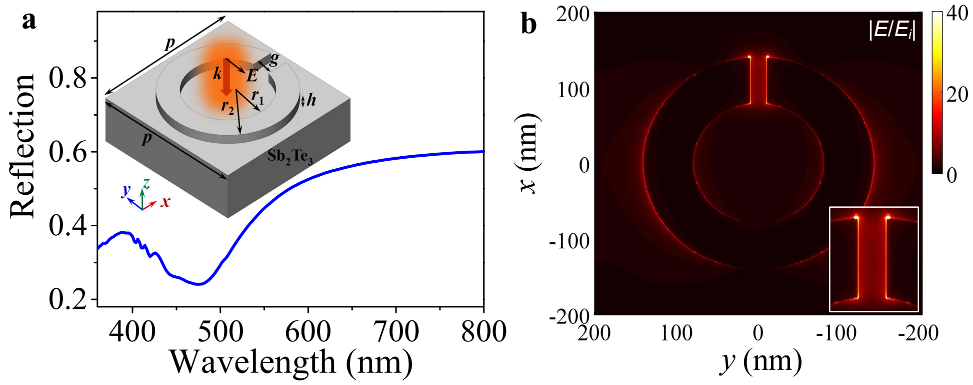

Fig. S10 Modified structure for improved electric field enhancement. a Reflection spectrum from the Sb_2Te_3 TI split-ring structure when the incident polarization is perpendicular to the gap. The inset shows the schematic of a periodic unit of Sb_2Te_3 split-ring array as an example for improving electric field enhancement. The thickness, inner radius, out radius, gap width and pitch of split rings are set as h=50 nm, r_1=80 nm, r_2=140 nm, g=20 nm and p=400 nm, respectively. **b** Distribution of electric field $|E/E_i|$ in a periodic unit of Sb_2Te_3 split-ring structure at the wavelength of 480 nm. The inset shows the electric field distribution in the split-ring gap. It is found that the electric field intensity can be enhanced by >1600 fold.

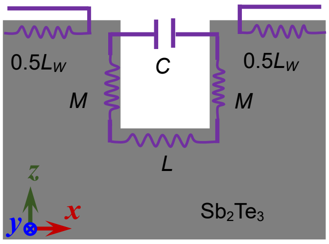

Fig. S11 MLC circuit model for MPR. MLC circuit model for theoretical analysis of MPRs in the Sb_2Te_3 TI nanogrooves.

Table S1. Fitting physical parameters of Drude and Tauc-Lorentz models for surface and bulk states of single-crystalline Sb_2Te_3 TI.

Physical parameters	Fitted values (units)
Drude surface layer thickness	2.6±0.5 (nm)
E_g (Tauc-Lorentz)	0.33 ± 0.09 (eV)
ε_∞ (Tauc-Lorentz)	3.28 ± 0.25
A (Tauc-Lorentz)	89.5 ± 10.2 (eV)
E_0 (Tauc-Lorentz)	1.39 ± 0.02 (eV)
D (Tauc-Lorentz)	1.40 ± 0.05 (eV)
$\varepsilon_{\infty,s}$ (Drude)	1
ω_p (Drude)	7.98 ± 0.91 (eV)
Γ_d (Drude)	0.16 ± 0.03 (eV)

Supplementary Methods

Preparation process of the Sb_2Te_3 microflake for TEM measurement

The preparation process for TEM sample of Sb_2Te_3 single crystal microflake can be described as follows. A copper microgrid with the carbon support film is used to prepare the TEM sample of Sb_2Te_3 material. The Sb_2Te_3 flakes are exfoliated from the Sb_2Te_3 single crystal and transferred onto a SiO_2 layer (100 nm thickness) on Si substrate. Then, a KOH solution with the concentration of 1-2 mol/L is dropped on the copper microgrid frontally covered on SiO_2/Si substrate with the Sb_2Te_3 flakes. Finally, the copper microgrid disengages from the substrate after the SiO_2 layer is etched, and then is submerged in isopropyl alcohol for several minutes. The TEM image of Sb_2Te_3 microflake and EDS mapping pictures are shown in Fig. 1b. The SAED pattern and high-resolution TEM image are plotted in Fig. 1c.

Spectroscopic ellipsometry measurement of optical constant for Sb_2Te_3 single crystal

The Sb_2Te_3 TI single crystal can be considered as a bulk insulator (or semiconductor) coated with an ultrathin Drude surface conducting layer[S3]. The relative permittivities of bulk and surface layers for the Sb_2Te_3 TI in the UV, visible and near-infrared ranges can be achieved by fitting the experimental data from spectroscopic ellipsometer with the Tauc-Lorentz and Drude models, respectively. The relative permittivity of bulk semiconducting layer can be mainly attributed to the interband transition, whose band structures can be described by the Kramers-Kronig equations[S4,S5]. Thus, the imaginary and real parts (ε_b'' and ε_b') of relative permittivities for the TI bulk can be expressed as

$$\varepsilon_b''(E)=\begin{cases}\dfrac{E_0AD\left(E-E_g\right)^2}{D^2E^2+\left(E^2-E_0^2\right)^2}\cdot\dfrac{1}{E}, & E>E_g\\ 0 & E\le E_g\end{cases} \tag{S1}$$

$$\varepsilon_b^{'}(E)=\varepsilon_\infty+\frac{2}{\pi}P\int_{E_g}^{\infty}\frac{\xi\varepsilon_b^{''}(\xi)}{\xi^2-E^2}d\xi, \qquad \text{(S2)}$$

where E_0, A, D and E_g represents the peak in joint density of states, absorption peak amplitude, broadening factor and bandgap energy, respectively. E stands for the energy of incident photons. ε_∞ is the relative permittivity of bulk semiconductor at high frequency, and P is the Cauchy principal part of the integral[S5]. The relative permittivities of surface conducting layer can be described as the Drude model: $\varepsilon_s(\omega)=\varepsilon_{\infty,s}-\omega_p^2/(\omega^2+i\Gamma_d\omega)$, where $\varepsilon_{\infty,s}$, ω_p and Γ_d are the relative permittivity at the high frequency, bulk plasma frequency and electron collision frequency of surface conductor, respectively[S6]. $\omega=2\pi c/\lambda$ is the angular frequency of incident light. By using the spectroscopic ellipsometer, we can obtain the wavelength-dependent ellipsometric angles Ψ and Δ (or I_S and Ic) with impinging light on the Sb_2Te_3 single crystal at the angle of 70°. Thus, the entire relative permittivities of Sb_2Te_3 single crystal can be experimentally achieved, as shown in Fig. 1d. The material model can be set as two layers: a thin surface layer with the Drude dispersion relation and a bulk substrate with the Tauc-Lorentz dispersion relation. By fitting the ellipsometry data in the software, we can obtain the entire relative permittivity of Sb_2Te_3 TI and individual relative permittivities of surface and bulk layers. From Fig. 1d, we can see that the fitting curves agree well with the experiment results. The achieved fitting parameters in the Tauc-Lorentz and Drude models are described in the Table S1. The value of χ^2 is 0.575, revealing the goodness of this fit. From Table S1, we find that the surface layer thickness is about 2.6 nm, which is similar to the reported 2.5 nm of 3D TI single crystal in the same family[S7]. The bulk band gap energy of 0.33 eV is in good agreement with the reported value of ~0.3 eV[S8,S9]. The relative permittivities of surface and bulk states are depicted in Figs. 1e and 1f, respectively.

MLC circuit model for MPRs in the Sb_2Te_3 nanogroove structures

The equivalent MLC circuit model is an effective method to theoretically analyze the MPR condition in grating structures[S10]. The MLC circuit model is employed to analyze the MPR response in TI nanostructures. As shown in Fig. S11, the two ridges at the top of Sb_2Te_3 nanogrooves can work as a capacitor. For the fundamental MPR mode, the capacitance in single current loop can be written as $C=c_1\varepsilon_0\varepsilon_d hl/d$ [S10]. Here, ε_0 and ε_d are the permittivity of vacuum and relative permittivity of dielectric (ε_d=1 for air) in the nanogrooves, respectively. l=1 denotes the nanogroove length along the y-axis direction in the structure. c_1 is a coefficient related with the charge distribution at the surfaces of nanogroove ridges. The TI ridges and bottom work as an inductor L containing ridge and kinetic inductances, which can be described as [S10]

$$L = \mu_0 hd - \frac{2h+d}{\varepsilon_0\omega^2\delta}\frac{\varepsilon'}{\varepsilon'^2+\varepsilon''^2}, \tag{S3}$$

where μ_0 is the permeability of vacuum. For simplicity, ε' (ε'') can be treated as the real (imaginary) part of Sb_2Te_3 relative permittivities in Fig. 1d. $\delta=\lambda/4\pi k_t$ is the penetration depth of light power in Sb_2Te_3. k_t is the extinction coefficient of Sb_2Te_3 and satisfies the relation: k_t $=\mathrm{Im}((\varepsilon'+i\varepsilon'')^{0.5})$. The surface inductance L_W and mutual inductance M of two adjacent circuits can be respectively described as[S10]

$$L_W = -\frac{0.5(p\text{-}d)}{\varepsilon_0\omega|\omega_s-\omega|\delta}\frac{\varepsilon'}{\varepsilon'^2+\varepsilon''^2}, \tag{S4}$$

$$M = \kappa\sqrt{L_W L}, \tag{S5}$$

where κ is the coupling efficient of two circuits. ω_s is the plasmon frequency of the nanogroove grating, which can be expressed as $\omega_s=2\pi c/p$ for the normally incident light. The total impedance of the circuit in the nanogrooves can be written as $Z=i[\omega L+\omega M-1/(\omega C)]$. According to the resonance condition of Z=0, the resonance wavelength can be written as[S10]

$$\lambda_M = 2\pi c\sqrt{(L+M)C} \tag{S6}$$

These equations can be used to theoretically calculate the MPR wavelengths in the nanogroove grating. As depicted in Fig. 3b, the theoretical calculations agree well with the experimental

and simulation results. Here, the coefficients c_1 and κ can be set as 0.82 and 0.25, respectively.

References

S1. Zybała, R. *et al.* Synthesis and characterization of antimony telluride for thermoelectric and optoelectronic applications. *Arch. Metall. Mater.* **62**, 1067-1070 (2017).

S2. Sarychev, A., Shvets, G. & Shalaev, V. Magnetic plasmon resonance. *Phys. Rev. E* **73**, 036609 (2006).

S3. Zhang, H., Liu, C., Qi, X., Dai, X., Fang, Z. & Zhang, S. Topological insulators in Bi_2Se_3, Bi_2Te_3 and Sb_2Te_3 with a single Dirac cone on the surface. *Nat. Phys.* **5**, 438-442 (2009).

S4. Palik, E. D. *Handbook of Optical Constant of Solids II*. Academic Press, 151-166, 1991.

S5. Jellison, G. & Modine, F. Parameterization of the optical functions of amorphous materials in the interband region. *Appl. Phys. Lett.* **69**, 371 (1996).

S6. Ou, J., So, J., Adamo, G., Sulaev, A., Wang, L. & Zheludev, N. Ultraviolet and visible range plasmonics in the topological insulator $Bi_{1.5}Sb_{0.5}Te_{1.8}Se_{1.2}$. *Nat. Commun.* **5**, 5139 (2014).

S7. Xia, B., Ren, P., Sulaev, A., Liu, P., Shen, S. & Wang, L. Indications of surface-dominated transport in single crystalline nanoflake devices of topological insulator $Bi_{1.5}Sb_{0.5}Te_{1.8}Se_{1.2}$. *Phys. Rev. B* **87**, 085442 (2013).

S8. Jiang, Y., Wang, Y., Chen, M., Li, Z., Song, C., He, K., Wang, L., Chen, X., Ma, X. & Xue, Q. Landau quantization and the thickness limit of topological insulator thin films of Sb_2Te_3. *Phys. Rev. Lett.* **108**, 016401 (2012).

S9. Ando, Y. Topological insulator materials. *J. Phys. Soc. Jpn.* **82**, 102001 (2013).

S10. Guo, Y., Shuai, Y. & Tan, H. Mechanism of polaritons coupling from perspective of equivalent MLC circuits model in slit arrays. *Opt. Express* **27**, 21173-21184 (2019).
